# ASO Author Reflections: Objective Outcome Measure of Upper Limb Function Following Axillary Lymph Node Dissection and Sentinel Lymph Node Biopsy

**DOI:** 10.1245/s10434-023-13984-5

**Published:** 2023-07-23

**Authors:** Nur Amalina Che Bakri, Richard M. Kwasnicki, Emmanuel Giannas, Luqman Tenang, Naairah Khan, Catharina Moenig, Zoha Imam, Kieran Dhillon, Hutan Ashrafian, Ara Darzi, Daniel R. Leff

**Affiliations:** 1 Academic Surgical Unit, Department of Surgery and Cancer, Imperial College London, London, UK; 2 Imperial College Healthcare NHS Trust, St. Mary’s Hospital, London, UK

## Past

Breast and axillary surgery can impair arm movement, restrict physical function and limit quality of life.^1^ The misperception that impairments resolve swiftly without intervention has led to a diminished emphasis on monitoring upper limb disabilities, particularly using objective measures.^2^ Instead, subjective measurements such as quality of life questionnaires and self-reported outcomes have previously been used to assess post-operative morbidity. These tools are vulnerable to bias, and do not objectively assess functional morbidity associated with sentinel lymph node biopsy (SLNB) and axillary lymph node dissection (ALND).^3^ Objective measures, such as goniometry and tape arm measurements, rely on the operator’s skills and may be problematic owing to inter-observer variation.^4^ A recent systematic review emphasized the need for quantitative and validated outcome measures, as well as the lack of standardization in the measurement of upper limb morbidity.^1^

## Present

We previously validated the use of wearable activity monitors (WAMs) in the peri-operative period to objectively quantify physical activity (PA) levels following breast surgery.^5^ An objective, longitudinal measurement of PA during the post-operative period would provide more information to the patient and surgeon about the upper limb and total activity levels associated with their surgery. WAMs could reduce measurement and operator variability while monitoring post-operative upper limb activity levels in a non-invasive and unbiased manner. WAMs objectively demonstrate return of PA to pre-operative baseline and differentiate recovery across different surgical treatments. This study investigated the use of WAMs to compare physical recovery between ALND and SLNB.^6^ Based on the analysis of 56 patients (SLNB 35, ALND 21), the findings demonstrated that ALND resulted in a significantly lower PA level in week 2. In addition, physical restrictions following SLNB persisted for 2 weeks following surgery. This is an important finding because it suggests that even de-escalated axillary procedures are associated with prolonged morbidity.

## Future

In the future, WAMs could be implemented routinely to map long-term physical recovery following breast and axillary surgery. WAMs might be used in conjunction with structured exercise regimens to enable healthcare providers to track patients’ levels of activity and offer tailored, real-time feedback depending on PA levels. WAMs can deliver behavioral signals or motivational messages to patients that encourage them to perform the prescribed exercises. As evidence begins to strengthen the relationship between PA and survivorship after breast surgery, these strategies are pertinent.^7^ WAMs have the potential to improve patient satisfaction, cost efficiency, and functional outcomes, if they are utilized appropriately.
